# Asymmetric Additions Empowered by OrganoCatalysts, Metal Catalysts, and Deep Natural Eutectic Solvents (NADES)

**DOI:** 10.1021/acs.joc.4c00334

**Published:** 2024-05-02

**Authors:** Elisabete P. Carreiro, Gesine J. Hermann, Hans-Jürgen Federsel, Anthony J. Burke

**Affiliations:** † LAQV-REQUIMTE, Institute for Research and Advanced Studies, University of Évora, Rua Romão Ramalho 59, 7000-671 Évora, Portugal; ‡ Chiratecnics, PO Box 59, Rossio, Évora, 7006-802, Portugal; § School of Sciences and Technology, Fase III, Laboratory 010, University of Évora, Rua Romão Ramalho 59, 7000-671 Évora, Portugal; ∥ RISE Research Institutes of Sweden, Box 5607, SE-114 86 Stockholm, Sweden; ⊥ Faculty Pharmacy, University of Coimbra, Pólo das Ciências da Saúde, Azinhaga de Santa Comba, 3000-548 Coimbra, Portugal; # Centro de Química de Coimbra - Institute of Molecular Sciences (CQC-IMS), Departamento de Química, Faculdade de Ciências e Tecnologia, Universidade de Coimbra, 3004-535 Coimbra, Portugal; ¶ Center for Neurosciences and Cellular Biology (CNC), Polo I, Universidade de Coimbra Rua Larga Faculdade de Medicina, Polo I, 1°andar 3004−504, Coimbra, Portugal

## Abstract

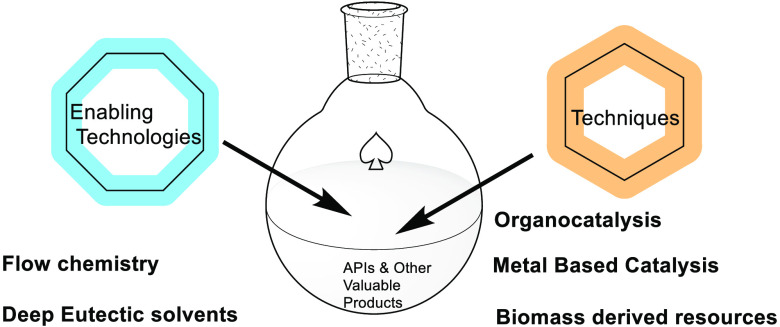

This article is a history of an industrial–academic partnership that started almost two decades ago and details the evolution of a relationship between a small academic research group and a spin-out company located in Portugal. Their activities have ranged from the development of new metal-based catalytic systems for asymmetric epoxidations, allylic alkylations, and arylations to the development of novel cinchona-based organocatalysts for asymmetric hydrosilylations and Michael additions. Current common interests are centered on the development of novel chiral Natural Deep Eutectic Solvent systems, which they are investigating in different types of reaction systems.

## Introduction

In 2001, Anthony Burke was awarded a grant to study some new sustainable processes, leading to important enantiomerically enriched target compounds. One of the principal objectives was the development of a catalytic asymmetric epoxidation process of nonfunctionalized alkenes with methylrhenium(VII)trioxide (MTO). Some years before the groups of Sharpless and Herrmann showed that certain aromatic amines like pyridine^1^ and pyrrazole^2^ could accelerate this reaction, and due to the efficiency of this method, it was of interest to develop an asymmetric catalytic method.

## Trost’s Cyclohexane-bis-pyridinamide

**Figure 1:**
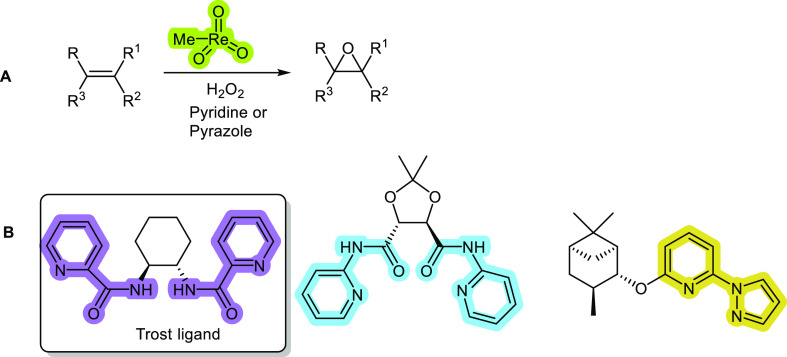
**A**. The MTO-catalyzed epoxidation of olefins. **B**. The ligands prepared and investigated in the MTO-catalyzed asymmetric epoxidation reaction.

Our mission was to develop chiral pyridine ligands that could bind to the Re center. We first reported a novel efficient method of epoxidizing nonfunctionalized alkenes using MTO with urea–hydrogen peroxide and heterocyclic amine catalysts (A, Figure 1).^3^ This was very successful and paved the way for the development of an asymmetric catalytic version. A number of different chiral pyridine ligands were synthesized in the group, but unfortunately, after exhaustive screening studies, the highest enantioselectivity achieved was only 12% ee, using bis-pyridinamide and a menthol-pyridine-pyrazole ligand (B, Figure 1).^4^ Interestingly, one of the ligands was the Trost cyclohexane-bis-pyridinamide (7% ee), which had previously been very successfully used in the Molybdenum Asymmetric Allylic Alkylation (MoAAA) reaction.^5^ For this task, we developed a novel method to prepare this important ligand, which was very efficient and could be scaled-up to a 1 kg scale. It must be noted that around that time, Merck had developed a method for the synthesis of a CCR5 antagonist candidate that relied on Trost’s MoAAA as a key step (this was later used as a microbicide known as CMPD 167).^6^ Several important mechanistic studies were conducted, revealing the interactions between the Mo and the ligand. Ultimately, considering the importance of this ligand from a commercial standpoint, we put together a commercialization plan with the help of our university, which resulted in the creation of Chiratecnics. The technology for the synthesis of the Trost ligand was later transferred to Chiratecnics for commercialization and is still available from the company.

## Tartaric Acid Based Building Blocks-Chiral Ligands for Asymmetric Catalysis

**Figure 2:**
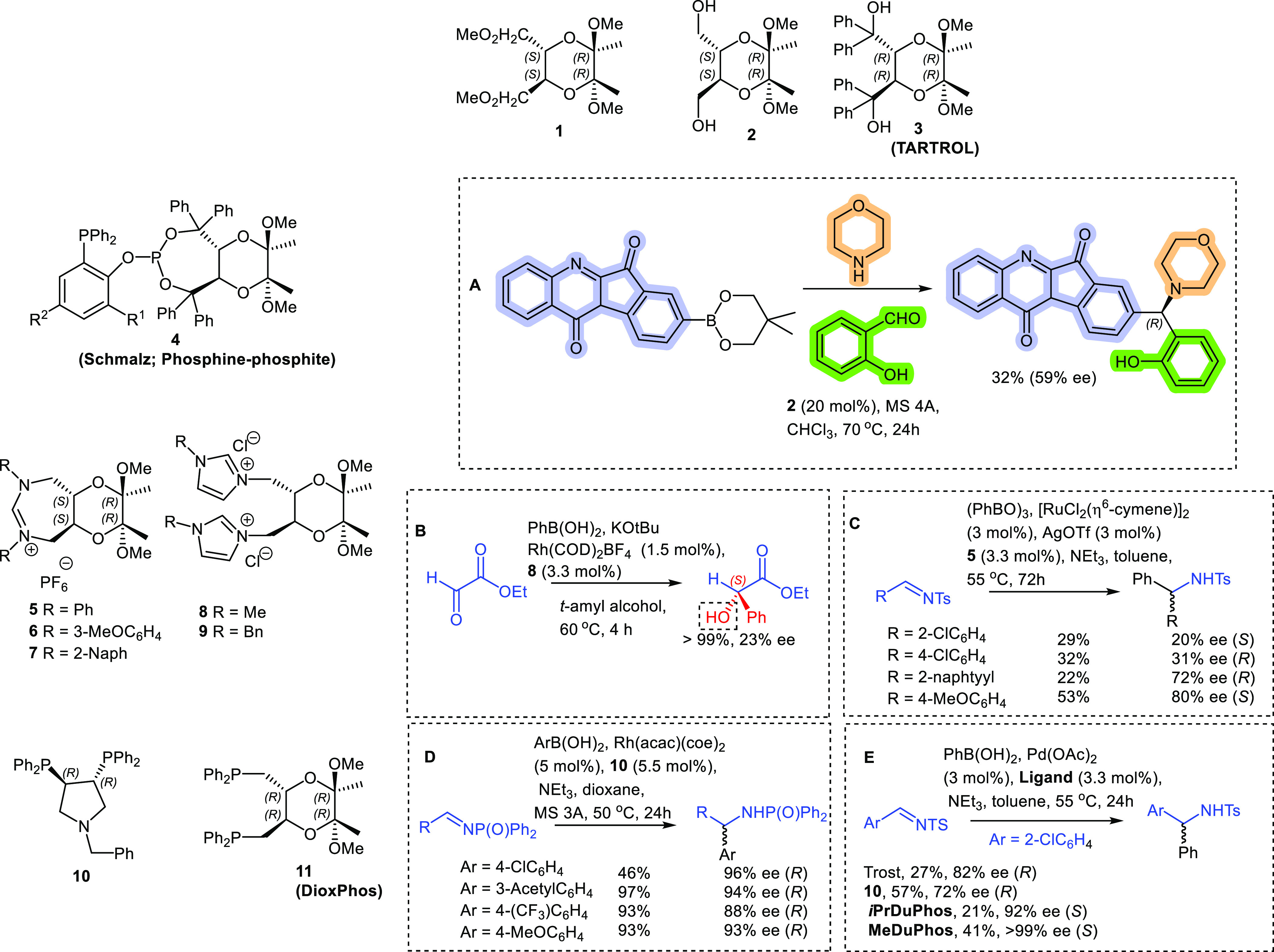
Some key chiral ligands based on tartaric acid **A.** The asymmetric Petasis reaction. **B.** Asymmetric Rh-catalyzed arylation of activated aldehydes. **C.** Asymmetric Rh-catalyzed arylation of tosyl-activated imines. **D.** Ellmans’s method for catalytic arylation of diphenylphosphinoyl imines. **E.** First report on the use of Pd-phosphine catalysts for imine arylations with arylboronic acids.

Chiratecnics subsequently developed a small portfolio of chiral ligands and building blocks derived from renewable resources, which were mostly amines and phosphines, and became a vendor to several international chemical companies. Some of these compounds are of interest to the synthetic medicinal chemist, like dimethoxy-2,3-dimethyl-1,4-dioxane-5,6-dicarboxylate (**1**),^7^ which was used as a starting material for many important biologically active compounds,^8^ such as (+)-aspicilin (Figure 2). It can be efficiently reduced to the diol derivative (**2**),^9^ which has had use in asymmetric catalysis,^10^ and notably for some catalysts developed by Johnson-Matthey.^11^ This is commercialized by Chiratecnics under the brand-name *Diolane*. As a further development, in 2021, Burke and co-workers showed that *Diolane* could be used successfully in the asymmetric catalytic Petasis reaction, affording enantioselectivities of 59% ee in a test reaction (**A**, Figure 2).^10^

In 2012, Marques and Burke reported the use of chiral di-NHC (*N*-heterocyclic carbenes)-based ligands (**5**)–(**9**) in the Rh(I)-catalyzed asymmetric catalytic arylation of ethyl glyoxylate using organoboron reagents (**B**Figure 2).^12^ The enantioselectivities were low (21–28% ee), even upon tuning to incorporate bulkier substituents on the NHC unit. The yields were generally excellent with the monodentate ligands and with the bidentate ligand (**9**).^13^ To show the industrial potential of this procedure this invention was claimed as Portuguese patents.^14,15^

The NHC (**9**) was used successfully in Ru-catalyzed arylations using phenylboroxine (**C**Figure 2), affording enriched (*S*)-tosylamine products in moderate yields (29–53%) and enantioselectivities of 29–80% ee.^16^

Chiratecnics has become a very proficient producer of the C_2_-symmeric phosphines, which are mostly useful for asymmetric hydrogenations. For instance, the Beren’s ligand (**3**) (Figure 2; commercialized under the brand name DioxPhos by Chiratecnics)^17^ is derived from *Diolane* (besides this ligand, the company also commercializes the DeguPhos ligand (**4**); see below for further details).

Diacetal (**1**) can be transformed to the bis-tertiary alcohol TARTROL (**3**) via a method originally developed by Berens and co-workers^16^ and subsequently used by Schmaltz and co-workers to form modular phosphine-phosphite ligands (**4**), which were successfully used in Cu-catalyzed 1,4-additions to enones affording a highest enantioselectivity of 84% ee.^18^

DeguPhos (**10**) was originally developed by Degussa (now Evonik) for asymmetric hydrogenations.^19,20^ This is considered a very rigid ligand and can explain the good results that are obtained with this ligand in asymmetric hydrogenations. In 2005, Ellman showed the utility of this ligand for asymmetric Rh(I)-catalyzed arylations of activated imines to afford useful chiral amines with high enantioselectivities (**D**Figure 2).^21^ In fact, Marques and Burke were the first to use chiral phosphine ligands with Pd catalysts for the arylation of activated imines,^22^ achieving yields of up to 77% and enantioselectivities of over 99% ee (**E**Figure 2). However, one of the diphosphine ligands screened was DioxPhos (**11**), which is a ligand commercialized by Chiratecnics with an interesting history in the field of asymmetric hydrogenation, having been used by Zhang and co-workers (and commercialized by ChiralQuest) for the Rh(I)-catalyzed hydrogenation of enamide substrates that afforded amino alcohols with enantioselectivities of between 94 and 99% ee.^23^ DioxPhos afforded the corresponding diarylamine in a yield of 77% and an enantioselectivity of 42% (unoptimized result).

## Cinchona Catalysts – For Asymmetric Organocatalysis

### Cinchona-Pyridinamide – Asymmetric Hydrosilylations for Single Enantiomer Amines

**Figure 3:**
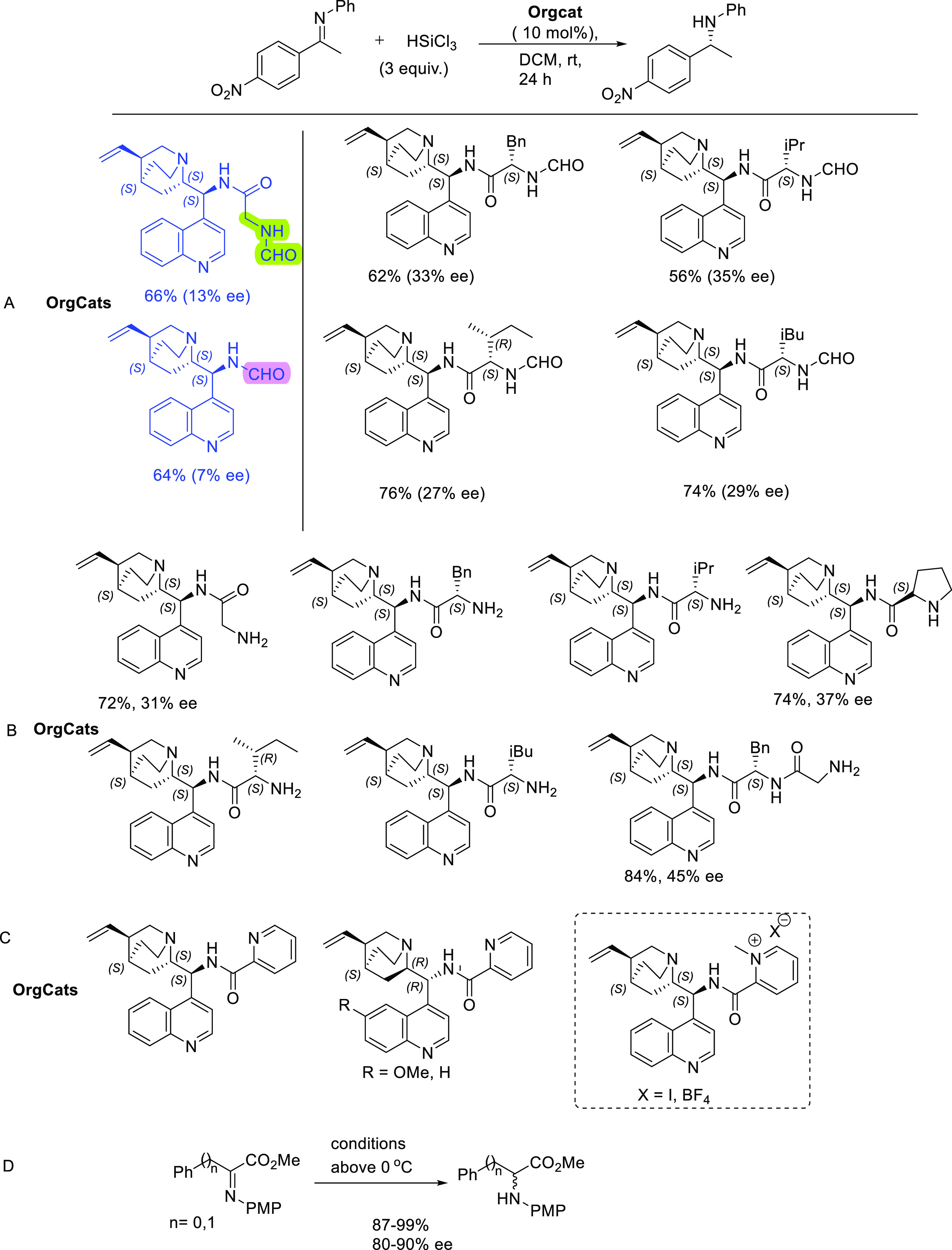
**A.** Hydrosilylation with cinchona-amino acid formamides. **B.** With cinchona-amino acids and peptides. **C.** Cinchona-picolinamides including the quarternary ammouium salts (in box). **D**. Some key asymmetric hydrosilylation reactions.

Later, Burke’s group developed novel cinchona-formamide, -amino acid, and -pyridinamide (for picolinamide) catalysts that were used for asymmetric hydrosilylation of aromatic imines. This is a very important reaction, leading to key API intermediates under nonhazardous and sustainable conditions. In a 2014 report, Burke and co-workers prepared a family of cinchona-formamide, -amino acid, and -peptide hybrids that were tested in the asymmetric hydrosilylation reaction.^24^ Some cinchona-amino-formamides were synthesized and tested in a benchmark reaction with *p*-nitroacetophenone (**A**Figure 3). Formamides have previously been successfully used in the hydrosilylation reaction.^25^ Unfortunately, despite our best efforts, the results were only moderate (with a best enantioselectivity of only 35% ee).

**Figure 4:**
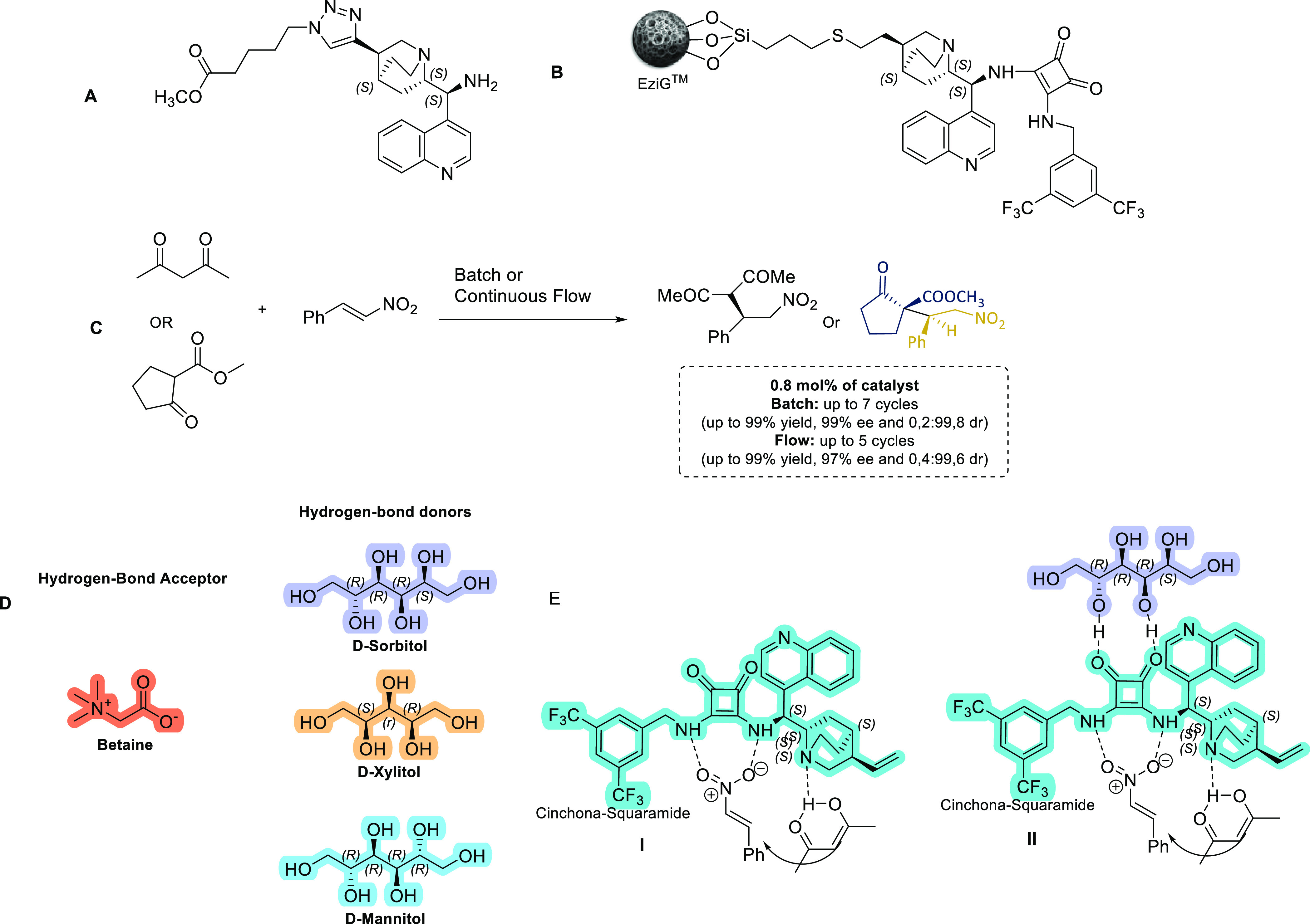
Some key highlights from activities in the field of organocatalysis. **A.** A novel 1,2,3-triazole containing amino-cinchona catalyst for solid immobilization. **B.** First example of a Cinchona-Squaramide catalyst immobilized to a Controlled Porous-Glass Bead (CPGB) support. **C.** Benchmark Michael reaction explored with CPGB-immobilized Cinchona-Squaramide catalyst and in NADES systems. **D.** NADES systems used for the organocatalyzed Michael reactions. **E.** Transition-state models for the Michael reactions (I: standard transition-state model with NADES components; II: speculative transition-state model with NADES components).

A series of cinchona-amino acid and peptide catalysts was then prepared and tested in the same reaction. Again, the enantioselectivities were only moderate (**B**Figure 3). Only the best results are shown in Figure 3, in which the highest ee of 45% was obtained. Unfortunately, other isomeric cinchonas, like cinchonine or quinidine, were not tested. Since the pioneering work of Matsumura in 2006 on the use of picolinamides, many other groups became interested in this method for the trichlorosilane hydrosilylation of ketimines,^26^ including ourselves, for which we developed (in collaboration with the Benaglia group) and applied cinchona-picolinamide catalysts for this reaction^27^ (**A**Figure 3). These showed excellent results, in terms of yields and enantioselectivities for the hydrosilylation of aromatic *N*-arylimines.^27^ Some important high-yielding and enantioselective reactions were conducted using the imines derived from methyl benzoyl formate and methyl benzoylacetate (**D**Figure 4). These reactions were showcased in a formal synthesis of the Alzheimer’s drug, Rivastigmine.^28^ Unfortunately, high enantioselectivities could not be achieved using the *N*-benzyl-substituted imine substrates, and the best enantioselectivity observed was 32% ee.^29^ Currently, the Burke group, in collaboration with the group of Natalia Cordeiro (chemistry department, University of Porto), are looking into the mechanism of the reaction, particularly from an enantioselectivity point of view.^30^

These groups developed heterogeneous versions of the reaction, which were also applied in continuous flow.^31,32^ These compounds have now become part of Chiratecnics’ organocatalyst portfolio.

Burke and Barrulas then developed a second-generation pyridinium-based salt version of this catalyst,^26,32^ which showed some improvements (**C**Figure 3) and could also be immobilized to solid supports, and which was patented by the University of Évora.^32^ Considering Chiratecnics’ experience and track record in the field of chiral technologies, the University of Évora entered a commercialization agreement with Chiratecnics to explore the commercialization of this technology. However, later studies supported by Chiratecnics showed that it was difficult to reproduce the synthesis of these compounds.

### Cinchona-Squaramides: Introducing C–C Bonds Enantioselectively Based on Porous Bead Supports

In more recent times, Chiratecnics, in collaboration with the University of Évora, has developed immobilized versions of the Rawal Cinchona-Squaramide catalyst, via immobilization to special controlled porous glass beads known as EziG (developed by EnginZyme in Sweden), which were successfully applied to Michael addition reactions in both batch and flow chemistry.^33^ The immobilization of organocatalysts to supports is an important undertaking considering that in many cases, the catalysts are expensive (due to lengthy reaction steps and the high loads that are required (10–20 mol %)), and from an industrial point of view, this approach makes sense to cut costs.^34^

The 1,2,3-triazole linker (derived from the highly efficient copper-catalyzed azide alkyne reaction^35^) has been used frequently for the immobilization of organocatalysts to various supports and used subsequently in continuous flow procedures. Based on previous work by Benaglia and co-workers,^36^ who developed some cinchona-amino catalysts containing a 1,2,3-triazole tether, we developed in collaboration with Chiratecnics the amino-cinchonidine-triazole methyl ester (**A**Figure 4) that we wished to functionalize with the squaramide unit and immobilize to a cellulose support. After much experimentation without any success, we changed strategy to immobilizing the Rawal catalyst to the EziG beads using the *Thiol–Ene* methodology originally developed by Barrulas and Burke to immobilize their pyridinium based salts to inorganic supports,^32^ (and later used to immobilize the cinchona-picolinamide congeners to polymer supports^31^); this was in fact a great success. We could attach the Rawal catalyst to three types of EziG beads with variations in their surface properties, ranging from hydrophilic to hydrophobic (**B**Figure 4; it should be noted that the stereochemical configuration at C-9 in our paper was incorrectly indicated as *R*). To establish the feasibility of these catalysts, we used them in standard benchmark Michael additions. The reactions were conducted in batch and continuous flow modes, with two substrates: acetylacetone and methyl 2-oxocyclopentane (**B**Figure 4). Under batch conditions at enviable loads of 0.8 and 1.6 mol %, the Michael adducts were obtained in excellent yields and stereoselectivities (enantio- and diastereoselectivity). We could also reproduce these excellent results under continuous flow conditions using a small fixed-bed catalytic reactor. The immobilized catalysts could be recycled several times under batch conditions, and fresh substrates could be added repeatedly under continuous flow conditions.

### Asymmetric Organocatalytic Michael Additions in Natural Deep Eutectic Solvents (NADES)

Chiratecnics, in collaboration with the University of Évora and the Faculty of Science and Technology, New University of Lisbon, has also successfully demonstrated the efficacy and selectivity of this reaction in chiral Deep Eutectic Solvents (DESs), which can allow multiple catalyst recycling modes. Instead of using the standard choline chloride with urea and glycerol, we opted to use betaine (trimethylglycine) and some simple C5- and C6-sugars. We studied the benchmark Michael additions described above with the nonimmobilized Rawal catalyst, and obtained excellent results, in terms of yields, stereoselectivities, and recovery.^37,38^ We investigated three types of betaine-based DESs with three different sugars: d-sorbitol, d-xylitol, and d-mannitol (**D**Figure 4). We found that the d-sorbitol-based DES was the best performer, and we could carry out multiple cycles at catalyst loadings of 1 and 5% (up to 10 cycles in the case of methyl 2-oxocyclopentane-1-carboxylate, achieving a yield of 97%, ee of 93% and de of 97% on the 10th cycle). One of the problems encountered were oscillations in the enantioselectivities, which were attributed to competing transition states involving the catalyst and probably the sugar component from the DES (**E**Figure 4). We also obtained strong evidence to indicate that the sugar unit is involved, as one of the reactions without the Rawal catalyst was enantioselective (75% ee, using acetylacetone and nitrostyrene with sorbitol present). The Burke group in Coimbra is currently using these systems in multi-component reactions.

## Conclusions

In this Perspective, we have discussed in detail the scientific discoveries and breakthroughs that have been achieved through these promising academic–industrial partnerships in a small agricultural-tourism-based European Mediterranean country. The main thrust of this collaboration was in the field of asymmetric catalysis (asymmetric epoxidations, allylic alkylations, and arylations, to the development of novel cinchona-based organocatalysts for asymmetric hydrosilylations and Michael additions). The collaboration has been highly synergistic and inspiring, allowing both sides to develop and flourish. We expect this partnership to extend into the future.

## Data Availability

The data underlying this study are available in the published article.

## References

[ref1] Herrmann W. A.; Fischer R. W.; Marz D. W. Methyltrioxorhenium as Catalyst for Olefin Oxidation. Angew. Chem., Int. Ed. Engl. 1991, 30, 1638–1641. 10.1002/anie.199116381.

[ref2] Rudolph J.; Reddy K. L.; Chiang J. P.; Sharpless K. B. Highly Efficient Epoxidation of Olefins Using Aqueous H_2_O_2_ and Catalytic Methyltrioxorhenium/Pyridine: Pyridine-Mediated Ligand Acceleration. J. Am. Chem. Soc. 1997, 119, 6189–6190. 10.1021/ja970623l.

[ref3] da Palma Carreiro E.; Burke A. J; Curto M. J. M.; Teixeira A. J.R An efficient and selective method for the epoxidation of olefins using urea-hydrogen peroxide and methyltrioxorhenium (VII) (MTO) catalyst with heterocyclic aromatic amines. J. Mol. Catal. A: Chem. 2004, 217, 69–72. 10.1016/j.molcata.2004.03.036.

[ref4] Trost B. M.; Hachiya I. J. Asymmetric Molydenum-Catalyzed Alkylations. J. Am. Chem. Soc. 1998, 120, 1104–1105. 10.1021/ja973298a.

[ref5] Barrulas P. C.; Genoni A.; Benaglia M.; Burke A. J. Cinchona-derived picolinamides: organocatalysts for stereoselective imine hydrosilylation. Eur. J. Org. Chem. 2014, 2014, 7339–42. 10.1002/ejoc.201403180.

[ref6] Yasuda N. CCR5 receptor antagonist. In The Art of Process Chemistry, Yasuda N., Ed.; Wiley-VCH: Weinheim, 2011, pp 45–75.

[ref7] Barros M. T.; Burke A. J.; Maycock C. D. The alkylation of a novel acetal derived from (2*R*,3*R*) - (+)-tartaric acid: An unexpected rearrangement. Tetrahedron Lett. 1999, 40, 1583–1586. 10.1016/S0040-4039(98)02650-1.

[ref8] Ley S. V.; Polara A. A Fascination with 1,2-diacetals. J. Org. Chem. 2007, 72, 5943–5959. 10.1021/jo0703451. 17430000

[ref9] Barros M. T.; Phillips A. M. F. Synthesis of new chiral amines with a cyclic 1,2-diacetal skeleton derived from (2*R*,3*R*) - (+)-tartaric acid. Molecules 2006, 11, 177–196. 10.3390/11020177. 17962789 PMC6148656

[ref10] Brandão P.; Marques C.; Pinto E.; Pineiro M.; Burke A. J. Petasis adducts of tryptanthrin – synthesis, biological activity evaluation and drug-likeness assessment. New. J. Chem. 2021, 45, 14633–14649. 10.1039/D1NJ02079J.

[ref11] Hems W. P., Grasa G. A. Biphosphine Ruthenium Complexes with Chiral Diamine Ligands as Catalysts. WO2005007662A2, 2005.

[ref12] Marques C. S.; Burke A. J. Expeditious and novel synthesis of α-hydroxyesters via rhodium-NHC catalysed arylation of ethyl glyoxalate. Tetrahedron 2012, 68, 7211–7216. 10.1016/j.tet.2012.05.129.

[ref13] Marques C. S.; Burke A. J. Enantioselective catalytic synthesis of ethyl mandelate derivatives using Rh(I)-NHC catalysts and organoboron reagents. Tetrahedron: Asymmetry 2013, 24 (11), 628–632. 10.1016/j.tetasy.2013.04.011.

[ref14] Burke A. J., Marques C. S. Processo catalítico para a arilação de ésteres de glioxalato em α-hidroxi-ésteres com metais-carbenos *N*-heterocíclicos monodentados, PT106376, 2013.

[ref15] Burke A. J., Marques C. S. Processo catalítico para a arilação de ésteres de glioxalato em α-hidroxi-ésteres com metais-carbenos *N*-heterocíclicos bidentados, PT107753, 2014.

[ref16] Marques C. S.; Burke A. J. Chiral diphosphane- and NHC-containing ruthenium catalysts for the catalytic asymmetric arylation of aldimines with organoboron reagents. Eur. J. Org. Chem. 2012, 2012, 4232–4239. 10.1002/ejoc.201200556.

[ref17] Berens U.; Leckel D.; Oepen S. C. Transacetalization of diethyl tartrate with acetals of alpha-dicarbonyl Compounds: A Simple Access to a New Class of C_2_-Symmetric Auxiliaries and Ligands. J. Org. Chem. 1995, 60, 8204–8208. 10.1021/jo00130a019.

[ref18] Dindaroğlu M.; Akyol S.; Şimşir H.; Neudörfl J.-M.; Burke A. J.; Schmalz H.-G. TARTROL-derived chiral phosphine-phosphite ligands and their performance in enantioselective Cu-catalyzed 1,4-addition reactions. Tetrahedron: Asymmetry 2013, 24, 657–662. 10.1016/j.tetasy.2013.04.008.

[ref19] Nagel U.; Albrecht J. The enantioselective hydrogenation of *N*-acyl dehydroamino acids. Top. Catal. 1998, 5, 3–23. 10.1023/A:1019141717606.

[ref20] Burke A. J.; Federsel H.-J.; Hermann G. J. Recent advances in asymmetric hydrogenation catalysis utilising spiro and other rigid C-stereogenic phosphine ligands. J. Org. Chem. 2022, 87, 1898–1924. 10.1021/acs.joc.1c01571. 34570501

[ref21] Weix D. J.; Shi Y.; Ellman J. A. Diastereoselective and enantioselective Rh(I)-catalyzed additions of arylboronic acids to *N*-tert-butanesulfinyl and *N*-diphenylphosphinoyl aldimines. J. Am. Chem. Soc. 2005, 127, 1092–1093. 10.1021/ja044003d. 15669835

[ref22] Marques C. S.; Burke A. J. Catalytic enantioselective addition of phenylboronic acid and phenylboroxine to N-tosylimines: PdII and RhI catalysis. Eur. J. Org. Chem. 2010, 2010, 1639–1643. 10.1002/ejoc.200901139.

[ref23] Li W.; Waldkirch J. P.; Zhang X. Chiral C2-Symmetric Ligands with 1,4-Dioxane Backbone Derived from Tartrates: Syntheses and Applications in Asymmetric Hydrogenation. J. Org. Chem. 2002, 67, 7618–7623. 10.1021/jo020250t. 12398481

[ref24] Barrulas P. C.; Benaglia M.; Burke A. J. Synthesis of novel cinchona-amino acid hybrid organocatalysts for asymmetric catalysis. Tetrahedron: Asymmetry 2014, 25, 923–935. 10.1016/j.tetasy.2014.05.003.

[ref25] Malkov A. V.; Mariani A.; MacDougall K. N.; Kocovsky P. Role of noncovalent interactions in the enantioselective reduction of aromatic ketimines with trichlorosilane. Org. Lett. 2004, 6, 2253–2256. 10.1021/ol049213+. 15200333

[ref26] Onomura O.; Kouchi Y.; Iwasaki F.; Matsumura Y. New organic activators for the enantioselective reduction of aromatic imines with trichlorosilane. Tetrahedron Lett. 2006, 47, 3751–3754. 10.1016/j.tetlet.2006.03.122. 16898818

[ref27] Barrulas P. C.; Genoni A.; Benaglia M.; Burke A. J. Cinchona-derived picolinamides: effective organocatalysts for stereoselective imine hydrosilylation. Eur. J. Org. Chem. 2014, 2014, 7339–42. 10.1002/ejoc.201403180.

[ref28] Genoni A.; Benaglia M.; Mattiolo E.; Rossi S.; Raimondi L.; Barrulas P. C.; Burke A. J. Synthesis of an advanced precursor of Rivastigmine: Cinchona-derived quaternary ammonium salts as organocatalysts for stereoselective imine reductions. Tetrahedron Lett. 2015, 56, 5752–5756. 10.1016/j.tetlet.2015.08.086.

[ref29] Fernandes S. Development of an organocatalytic method for the enantioselective synthesis of Rivastigmine, MSc Thesis, University of Évora, 2016.

[ref30] Silva-Santos E.; Teixeira F.; Burke A. J.; Cordeiro M. N. D. S. Imine hydrosilylation: A theoretical validation through experimental results. In International Symposium on Synthesis and Catalysis 2023; University of Évora, Vol. 5–8, Sep. 2023, p P34.

[ref31] Fernandes S.; Porta R.; Barrulas P. C.; Puglisi A.; Burke A. J.; Benaglia M. Stereoselective reduction of imines with trichlorosilane using solid-supported chiral picolinamides. Molecules 2016, 21 (1–9), 1182 10.3390/molecules21091182. 27608000 PMC6274114

[ref32] Barrulas P. C., Burke A. J. Novel Picolinamide-Cinchona Organocatalysts and derivatives, US9844773B2, 2017.

[ref33] Amorim A. C.; Fonseca D. P.; Carreiro E. P.; Hermann G. J.; Federsel H.-J.; Burke A. J. Immobilization of Functionalized epi-Cinchonine Organocatalysts on Porous Glass-Beads: Application in Batch and Continuous Flow. Synlett 2022, 33, 1756–1762. 10.1055/a-1916-4858.

[ref34] Amorim A. C.; Burke A. J. Ch. 29 Supported Chiral Organocatalysts for Accessing Fine Chemicals. In Catalysis for a Sustainable Environment, Pombeiro A., Alegria E., Sutradhar M., Eds.; Wiley, 2024, pp 639–657.

[ref35] Amorim A. C.; Burke A. J. What is the future of click chemistry in drug discovery and development?. Expert Opin. Drug Discovery 2024, 19 (3), 267–280. 10.1080/17460441.2024.2302151. 38214914

[ref36] Porta R.; Benaglia M.; Coccia F.; Cozzi F.; Puglisi A. Solid supported 9-amino-9-deoxy-epi-quinine as efficient organocatalyst for stereoselective reactions in batch and under continuous flow conditions. Adv. Synth. Catal. 2015, 357, 377–383. 10.1002/adsc.201400821.

[ref37] Amorim A. C.; Fonseca D. P.; Carreiro E. P.; Ramalho J. P.; Duarte A. R.; Hermann G. J.; Federsel H.-J.; Burke A. J. Sustainable OrganoCatalyzed Enantioselective Catalytic Michael Additions in Betaine derived Deep Eutectic Solvents. SynOpen 2023, 07 (03), 374–380. 10.1055/a-2117-9971.

[ref38] Carreiro E. P.; Federsel H.-J.; Hermann G. J.; Burke A. J. Stereoselective Catalytic Synthesis of Bioactive Compounds in Natural Deep Eutectic Solvents (NADESs): A Survey Across the Catalytic Spectrum. Catalysts 2024, 14, 160 10.3390/catal14030160.

